# Supplementary material for: Are carbon emissions trading and green financial instruments synergistic? -Comprehensive quantitative research based on content analysis
Source: PLoS One. 2024 Mar 7;19(3):e0298601. doi: 10.1371/journal.pone.0298601 (PMC10919601; doi:10.1371/journal.pone.0298601)
Supplement: S1 Data — (ZIP) [file pone.0298601.s001.zip › Data/地级市Z_F工作报告.zip!/╣π╢1⁄2/╔╟═╖╩╨/└┤╫╘╣1⁄2╓┌║┼í╛┬φ┐╦╩2╛▌═°í┐_2009.docx]

各位代表：

　　我代表市人民政府向大会作工作报告，请予审议，并请政协各位委员和其他列席人员提出意见。

　　一、2008年工作回顾

　　2008年是我市面对各种挑战、实现新的突破的一年。在省委、省政府和市委的正确领导下，市政府坚持以邓小平理论和“三个代表”重要思想为指导，深入贯彻党的十七大、省委十届三次全会、市委九届四次全会精神，全面落实科学发展观，解放思想，奋力开拓，积极应对全球金融危机的冲击和国内外经济形势的急剧变化，努力化解各种困难和矛盾，经济实现平稳较快发展，城乡建设出现可喜变化，社会保持安定有序，全年实现生产总值974.78亿元，增长10.5%，增速自1999年来首次高于全省水平；地方财政一般预算收入51.22亿元，增长20.6%。

一年来，我们主要做了以下工作：

（一）推动产业结构优化升级。落实各项扶持工业企业发展措施，工业经济进一步壮大，完成规模以上工业总产值1320.66亿元，增长18%。支持民营经济加快发展，实现民营经济增加值589.46亿元，占GDP的60.5%；民营投资额占全市投资额55.3%；5家企业进入全省民营百强企业行列。积极推动企业上市，1家企业通过上市审批，6家完成上市辅导。提高自主创新能力，推进产学研结合，完善科技创新体系，被确定为国家知识产权示范城市创建市。实现高新技术产品产值340亿元，增长22.7%。继续实施品牌战略，新获中国驰名商标2件，省著名商标61件，省名牌产品19个，地理标志保护产品1个。拓展建筑业国内外市场，完成施工产值168.7亿元。发挥传统商贸优势，完善多元化销售体系，社会消费品零售总额569.16亿元，增长20.3%。加强市场价格监管，物价涨幅得到有效控制，居民消费价格总水平上涨5.2%。改善金融业发展环境，金融机构本外币各项存贷款余额1365.86亿元和426.74亿元，分别增长17.5%和6.4%。积极推进市商业银行重组和农村信用社改革试点工作。大力发展滨海特色旅游，旅游业总收入67.89亿元，增长10.5%。加强现代信息服务业建设，信息化建设进入全国50强，实现本地固定电话号码升8位。稳定房地产市场，加快发展现代物流、文化创意产业。

　　（二）加快重大项目规划建设。完成全社会固定资产投资额261.36亿元，增长26.3%。100个重点建设项目年度完成投资额77.74亿元。三大经济带规划建设不断推进，东部城市经济带完成立项等前期审批手续，新津启动区项目完成水利工程初步设计和投资主体招标；工业经济带深圳龙岗（汕头潮南）产业转移工业园开工建设；生态经济带规划方案编制完成。广澳港区2号泊位建成试投产，防波堤获省立项，列入交通部“十一五”调整规划，《汕头港总体规划补充报告》通过审查。国道324线机场段工程竣工。加快建设华能海门电厂、国道324线潮阳路段大修工程，开工建设南澳大桥，推进汕揭梅高速公路汕头段、厦深铁路汕头段、疏港铁路、苏埃通道等重大项目的前期工作。完成牛田洋海堤、秋风岭水库、东里桥闸建设工程和省人大小型水库除险加固议案工程，加快实施农村机电排灌议案工程，一批城乡防灾减灾水利工程加快推进。新一代多普勒天气雷达系统和防灾减灾指挥中心建成投入使用。

　　（三）加强城市规划建设管理。积极参与粤东城镇群协调发展规划编制，从城市规划层面确立汕头市在粤东四市的中心地位。加快城市规划编制力度，推进全市总体规划修编和市区分区规划编制，城市控制性详细规划覆盖率70%，中心镇总体规划100%。多渠道筹措资金，全力推进一批市政道路建设，中山东路、海滨路、天山北路、金砂路东延路段、中泰立交桥首层道路等一批工程建成投入使用。加快金凤路桥建设，续建汕樟北路（金平段）、黄山路。抓好公路危桥排查整治，完成迴澜桥改造工程。开展道路运输市场秩序专项整治，摩托车治理和出租车市场整治初见成效。启用汽车客运中心站，撤销一批临时客运站配客点，运输市场秩序得到改善。加大公交投入，新开通公交线路8条，新增公交车辆84辆，市北区全面实施大货车禁限行，“出行难”问题逐步解决。出台城市道路停车泊位管理规定，规划设置公共停车泊位4669个，“停车难”有所缓解。加大创建国家园林城市力度，新增绿地面积30万平方米。规划建设城市景观照明工程，城市灯饰夜景格局初步形成。开通“12319”城市管理服务热线，持续开展市容环境综合整治，加大城市管理执法督察，城市管理长效机制逐步健全。加快一批环保硬件设施建设，龙珠水质净化厂技改和扩建项目建成投入使用，清源水质净化厂一期、雷打石生活垃圾填埋场建设完成，北轴污水处理厂、汕头（粤东）环境监控与应急指挥中心动工建设，龙湖沟、新河沟、三脚关沟完成整治。完成全国第一次污染源普查全面普查阶段工作。有效清除饮用水源保护区污染隐患。

　　（四）进一步深化改革开放。成功举办首届粤东侨博会暨经贸洽谈会，签约、开工、投产一批项目，投资总额347.5亿元。赴香港、西班牙等地开展招商引资活动，外商直接投资项目和增资扩产项目增多，实际吸收外资1.94亿美元，增长13%。主动参与海峡西岸经济区建设，积极争取国家政策支持，汕头港被列为对台海上直航口岸，实现在省内对台海运直航首航。引导企业适应国家外贸政策调整，创立自主品牌，优化产品结构，开拓新兴市场，转变外贸增长方式。完成出口总额43.23亿美元，增长10.5%。优化口岸通关环境，新开通中东、地中海远洋航线，实现港口货物吞吐量2806万吨，增长21.9%；集装箱吞吐量71.93万标箱，增长21.1%。潮阳港对外开放运作，汕头机场对外临时开放申报工作取得新进展。继续深化体制机制改革。实现全市范围内城乡用电同网同价。污水处理、垃圾处理收费制度进一步完善。加快市行政服务中心建设，新增进驻事项399项，比启用时增加近2倍；受理业务44796件，办结率97.8%。实现行政电子监察系统省、市、区三级联网，缩短审批时限，提高审批效率。出台加快国有企业改革总体方案，国有企业改革工作稳步推进。积极探索旧城改造新模式，推行以招拍挂方式确定项目建设主体，竣工新楼30.25万平方米。

　　（五）推进社会主义新农村建设。落实支农惠农政策，促进农业稳步发展，完成农业总产值99.81亿元，增长2.8%。加快农田水利基础设施建设，潮阳区列入国家农业综合开发项目区。特色农产品生产基地建设取得新进展，农业园区经济呈现良好发展势头，汕头潮南台湾农民创业园获得批准，新增国家农业标准化示范园2个、省级4个。扶持农民专业合作社和农业龙头企业发展，提高农业产业化水平，促进农民增收。扩大鲍鱼、对虾、青蟹等名优新品种养殖规模，提升渔业发展质量。国家级渔港海门中心渔港开工建设，达濠渔港列入国家一级渔港建设项目。

加强农村规划建设，完成扶持老区山区发展建设项目18个，正在建设20个；开工建设45个行政村113.7公里水泥路，全市通水泥路行政村达97%；增加农村客运站点112个。加快农村饮水安全工程建设，解决14.5万人“饮水难”问题。启动城中村和涉农社区供水直抄到户工程。加强村庄环境整治。开展创建林业生态区县活动。

　　（六）全面发展社会各项事业。认真落实市人大1号议案，促进教育全面协调发展。全面实施城乡免费义务教育，拨付免杂费补助金3.2亿元，免费提供1.2亿元教科书，发放贫困义务教育学生生活费补助3727万元。拆除中小学危房387幢12万平方米，维修校舍5.8万平方米，新建校舍24.34万平方米。加快普及高中阶段教育，投入2.6亿元，新、改、扩建校舍面积18.67万平方米，新增学位2万个。大力促进职业技术教育发展，汕头职业技术学院、鮀滨职业技术学校、林百欣科技中专等办学规模扩大，市第二技工学校升格为高级技工学校。完成特殊教育学校前期工作。完善农村中小学教职工保障制度，着手解决代课教师问题。健全疾控体系，新建潮南区疾控中心，改建澄海区、潮阳区和南澳县疾控中心，实施国家扩大免疫规划，重大传染病防控水平不断提高。推进公共文化设施建设，汕头戏曲学校易地重建，市博物馆新馆完成搬迁。蜈蚣舞等五个项目被列为第二批国家级非物质文化遗产名录。成功举办第三届国际潮剧节。圆满完成奥运火炬（汕头站）传递活动。我市运动员获得北京奥运会金牌2枚、残奥会铜牌1枚。深化双拥创建工作，密切军民军政关系。妇女儿童、残疾人、老龄、慈善和社会福利事业加快发展，外事、侨务、统计、人防、地震、档案、方志、民族、宗教等工作取得新成绩。

　　（七）着力完善多层次社会保障体系。加大促进就业力度，新增城镇就业岗位6.5万个，国有、集体企业下岗失业人员再就业9582人，城镇登记失业率3.1%，转移农村富余劳动力5.4万人。大力实施就业援助，帮扶“零就业”家庭810户。继续推进社保扩面，全市各项社会保险基金征集收入18.27亿元，增长26.1%。提高企业离退休人员基本养老金，月人均增加108元。建立被征地农民基本养老保障制度。新型农村合作医疗制度覆盖所有村居，参合率95.94%，住院补偿7.5万人次、1.83亿元。启动城镇居民基本医疗保险工作，登记参保人数达48.3万人。实施医疗救助制度，发放救助金470万元，救助8.7万人次。提高城乡居民最低生活保障标准和补助水平，受益群众8.88万人。高度重视危房处理应急工作，撤离安置危房户5075户，紧急安置501户市直管公房特危房住户。落实住房保障制度，筹集廉租公房474套2.7万平方米和租赁住房补贴资金410万元，对2995户城镇低收入住房困难家庭实施保障。实施15项惠民价格措施，切实减轻群众负担。中心城区居民人均可支配收入12542元，农村居民人均纯收入4885元，分别增长7%和6.6%。

　　（八）加强社会管理。建立全市应急联动体系，不断提高城市危机预警和应急管理能力，较好解决受极端天气影响的春运旅客滞留问题。进一步完善治安防控体系，实现全市110、119、122“三台合一”统一接处警。深入开展以潮南区为重点的严打整治专项行动，严厉打击各类违法犯罪活动。开展重信重访专项治理和大接访活动，妥善解决一批涉法涉诉、土地征用等信访突出问题。稳定防火安全和安全生产形势，事故总量持续下降，未发生重特大安全事故。进一步整顿和规范市场秩序，加大“打假”力度，2个省警示区域顺利摘帽。深化产品质量和食品安全专项整治成果，加强产品质量和食品安全监管，妥善处置三聚氰胺婴幼儿问题奶粉等突发性事件。加强动植物防疫检疫，强化农产品质量安全监管。落实最严格的耕地保护制度，查处违法违规用地3898宗、8458亩，拆除违法占地建筑1237亩，复耕复绿4049亩。顺利完成村（居）委会换届选举。全面完成省下达的年度人口计划指标，人口自然增长率5.67‰。落实殡葬目标管理责任考核制度，促进殡改工作发展。

　　（九）全民动员援助地震灾区恢复重建。派遣对口支援工作组，组织各职能部门开展实地调研，完成汶川县草坡乡恢复重建规划。确定援建项目73个3.6亿元，启动第一批项目12个，投资超过1亿元。开展救灾捐赠活动，筹集救助款2.1亿元，运送救灾物资及过冬物资价值3098万元。广泛动员社会力量，通过投资办实业、兴办公益事业等多种方式，协力做好对口支援工作。

　　（十）加强政府自身建设。出台健全完善科学民主决策制度规定、行政决策法律审查规定和行政决策听证规定，完善决策机制。推行行政执法责任制，全面落实行政首长问责制，加强行政执法监督，强化财政资金审计监督，规范行政执法行为。强化公共服务职能，全市财政用于民生民安支出增加8.61亿元，占增支总额的77.4%。坚决执行人大及其常委会决议，加强与人民政协联系。办结人大议案2件、建议70件、政协提案188件。提请市人大常委会审议法规草案2件，颁布政府规章8件、规范性文件21件。

各位代表！过去一年，我市经济社会发展取得的成绩，是省委、省政府和市委正确领导的结果，是各级人大及其常委会和人民政协监督支持的结果，是全市人民同心同德、奋力拼搏的结果，也是社会各方面支持和帮助的结果。在此，我代表市人民政府，向全市人民，向中央和省驻汕单位，向驻汕人民解放军、武警官兵、人民警察和各民主党派、人民团体、社会各界人士表示崇高的敬意！向关心和支持汕头建设的港澳台同胞、海外侨胞和国际友人表示衷心的感谢！

我们也清醒地认识到，我市经济社会发展仍存在不少困难和问题：一是经济总量偏小，产业结构层次偏低，工业产业集聚度不高，能带动、支撑全市经济社会持续发展的大项目少，现代服务业发展偏慢，辐射带动功能未能充分发挥。二是土地、资金、人才等瓶颈制约问题依然突出，企业自主创新能力不强，抵御风险能力和市场竞争力较弱，金融危机的影响正逐步显现。三是社会治安仍较严峻，生态环境保护需要加大力度，城市环境卫生需进一步综合治理。四是财政收入总量偏小，可支配财力非常有限，社会保障压力大，部分群众生活还比较困难。五是办事难、办事效率不高的问题尚未得到根治，行政效能建设亟待加强。对于这些问题，我们必须认真对待，采取强有力措施，扎实加以解决。

二、2009年经济社会发展主要目标和工作安排

　　2009年是我市全面实现“三年打基础”目标的一年，也是我市经济社会发展面临很大困难和严峻挑战的一年。当前，国际金融危机仍在不断扩散和蔓延，经济发展中的不确定因素明显增多，经济下行压力加大。我们必须把困难估计得更充分些。同时，也要充分认识到，为应对金融危机，中央和省相继出台了一系列扩大内需、促进经济增长的政策措施，实施积极的财政政策和适度宽松的货币政策，为我市加快城乡基础设施建设、产业结构调整升级、改善公共服务水平注入强大动力。国家在对台政策上给予我市大力支持，汕台实现海上直航，为汕头加强对台经贸合作，参与海峡西岸经济区建设，提高开放水平提供新的契机。省委、省政府加快经济特区发展的决定，为我们继续解放思想，深化改革开放，推动科学发展，增创特区新优势指明了方向。汕头目前已形成良好的发展态势，为我们迎难而上、加快发展奠定了坚实基础。对此，我们既要充分认识当前面临的困难，做好应对更加严峻局面的准备，更要坚定信心，化挑战为机遇，变压力为动力，危中寻机、危中奋起，牢牢抓住经济建设这个中心，审时度势，科学决策，周密部署，全力以赴抓好各项工作落实，努力保持经济社会平稳较快发展。

今年政府工作的指导思想是：以科学发展观统揽全局，全面贯彻落实党的十七届三中全会、中央经济工作会议、省委十届四次全会和市委九届六次全会精神，把加快发展保增长作为首要任务，以调整结构为主线，以投资拉动为重点，以改革开放为动力，以改善民生民安为落脚点，不断解放思想，创新体制，抢抓机遇，真抓实干，推动经济社会又好又快发展。

今年全市经济和社会发展的主要预期目标是：生产总值增长10.5%，工业总产值增长13%，单位生产总值能耗下降3%，二氧化硫新增控制在9.7%以内，化学需氧量削减7.4%，地方财政一般预算收入增长12.5%，全社会固定资产投资额增长16%，外贸出口总额增长5%，社会消费品零售总额增长16%，居民消费价格总水平涨幅控制在4%以内，城镇登记失业率控制在3.3%以内，城镇居民人均可支配收入增长7%，农村居民人均纯收入增长5%，人口自然增长率控制在8‰以内。

为实现上述目标，要做好九方面工作：

　　（一）以省扶持产业转移园区建设为契机，推动工业经济可持续发展

加快产业转移园区建设。全力加快深圳龙岗（汕头潮南）产业转移工业园区基础设施建设，吸引更多企业入园。继续申报认定省示范性产业转移工业园，积极争取省产业转移竞争性扶持资金支持。进一步完善园区规划，积极创新投融资体制，选择条件比较成熟的片区，作为工业经济带开发建设新的突破口。发挥海洋、侨乡、对台、民营优势，加大招商引资工作力度，承接先进地区产业转移，带动工业经济带全面启动发展。支持区县申报产业转移园区。

调整优化工业结构。大力实施“工业强市”战略，出台工业产业发展规划和工业布局规划，积极培育和发展石化、电力、船舶修造、装备制造等战略性产业，优化调整纺织服装、工艺玩具、食品加工、印刷包装等传统优势产业，重点发展电子信息、生物医药、新材料等高新技术产业。

推进产业集群发展。引导和促进优势产业和骨干企业集群发展，推进产业集聚和产业链延伸。选择具有一定规模优势的产业和特色产业，申报国家和省火炬计划产业基地。推动澄海玩具礼品、龙湖外砂潮式工艺毛织服装等产业集群升级示范区公共服务平台建设，支持特色产业集群发展壮大。发挥高新区政策优势，优化升级东片区产业结构，加快西片区开发建设，引导高新技术产业集聚，做强做大高新区。

　　（二）以重大项目建设为载体，提升城市集聚辐射功能

加快东部城市经济带规划建设。争取新津片区尽快启动建设，做好新溪、塔岗围片区水利工程勘察设计及规划，妥善解决用地用海历史遗留问题，争取早日启动，形成两头推进的格局。深化以牛田洋为起点的生态经济带规划编制。

加快交通基础设施建设。主动加强同国家和省有关部门的联系，争取更多项目进入计划“笼子”。推进汕头至揭阳、汕头至揭西、惠来至揭东、潮州至惠州高速公路汕头段建设。加快厦深铁路汕头段及联络线建设，抓紧阜鹰汕铁路、疏港铁路前期工作。完成国道324线潮阳路段大修工程，改造国道324线澄海路段，启动省道和惠公路大修工程。加快南澳大桥建设。继续做好苏埃通道等重大项目前期工作。推进广澳港区建设，力争3号泊位和5万吨级航道完工，启动10万吨级航道前期工作，加快防波堤项目和10万吨级集装箱码头前期工作。整治韩江内河航道。

加快能源、水利基础设施建设。推进华能海门电厂建设，争取500千伏送出工程上半年全线贯通，1、2号机组投产，抓紧3、4号机组前期工作。加快华润潮南风电项目建设。尽快建设丰盛海门电厂2台60万千瓦机组项目。加快南澳过海电缆前期工作。加快城乡防灾减灾重点水利工程建设。完成西港桥闸重建，动工建设莲阳桥闸，抓好下埔桥闸改造前期工作。继续开展水库除险加固，抓紧实施潮阳引韩供水工程和南澳引韩供水工程。

加快市政基础设施建设。继续抓好海滨路综合改造配套工程建设，推进金凤路桥、汕樟北路、金砂东路、泰山路、民族路、潮汕路等工程建设改造步伐。维修加固杏花桥，改建磊口大桥、和平大桥。继续推进第二过海水管工程。加快推进小公园历史风貌保护区、海滨路西段片区、新兴片区、梅溪河西岸及乌桥岛改造。加快南滨片区开发建设。

加快环保基础设施建设。继续推进“创模”整改达标工作。尽快开工建设雷打石生活垃圾焚烧发电厂、澄海区生活垃圾焚烧发电厂。加快北轴、南区、潮阳、潮南、南澳等一批污水处理厂及管网配套设施建设，力争完成汕头（粤东）环境监控与应急指挥中心建设。完成南区、潮阳、潮南生活垃圾卫生填埋场前期工作并启动建设。加强生态环境和生物多样性保护，推动南澎列岛海洋生态保护区晋升国家级自然保护区。加快沿海防护林体系建设。积极开展生态示范创建活动，加快海滨生态人居工程建设，构建适合人居的生态海滨城市。

　　（三）以推动城乡统筹发展为目标，加快农村改革发展步伐

　　加大农村制度创新力度。鼓励引导农民以各种形式流转土地承包经营权，发展适度规模经营。完善农业投入保障、农业补贴、农产品价格保护和农业生态环境补偿制度。鼓励金融机构加大对“三农”的信贷投入力度。进一步推进村务公开，完善民主管理，规范农村集体资产和财务管理。

　　加强农村基础设施建设。加强村庄整治规划建设，改善农村生产、生活环境。加大农村基础设施投入，建设一批农村文化、生活、环保设施。组织实施农田水利基本建设工程，打造一批现代标准农田。继续落实扶持老区山区项目。完成41个行政村107公里水泥路建设，实现全市行政村村村通水泥路目标。抓紧完成省人大农村机电排灌议案工程。加快海门中心渔港建设，开工建设达濠一级渔港，力争云澳渔港列入国家中心渔港建设项目。加快农村信息化建设。

　　积极发展现代效益农业。巩固和稳定粮食生产，积极推进省储备粮汕头直属库建设。大力调整优化农业结构，发展优质稻、蔬菜、水果、禽畜等十大农业产业。加快推进潮南台湾农民创业园、牛田洋现代农业生态示范区等十大现代农业园区规划建设。推广良种良法，推进设施农业、水稻生产机械化。加快农业科技创新和成果转化，推广特色种养技术。加快农业标准化建设。大力发展海洋经济，发展名优特色品种养殖和水产品精深加工，推动渔业增长方式转变。大力培育龙头企业和农村专业经济合作组织。深化畜牧兽医管理体制改革。加强动植物防疫检疫，强化农产品质量安全监管。

　　扎实推进农村劳动力转移。建立完善覆盖城乡的劳动力培训网络，实施“一户一技能”、农民工技能提升培训、智力扶贫等工程，促进农村劳动力技能提升。加强转移就业公共服务，完善农民工入户城镇政策体系。

　　（四）以生产性服务业为抓手，积极发展现代服务业

　　加快发展现代物流。加快海门港区煤炭转运码头建设，吸引煤炭、铁矿石等大宗货物从汕头港进出。积极寻求与大型航运企业合作，开辟更多国际航线。推进工艺玩具、纺织服装等专业批发市场的规划建设。

　　大力发展金融服务业。争取国内外银行保险机构、大型投资公司、风险基金到我市设立分支机构。加快市商业银行重组步伐。推进汕特农信联社改革。做好城信社和信托公司退市工作。积极探索农村金融组织试点，加快政策性农业保险制度建设。

推进新兴服务业发展。引导、扶持建设技术、信息、培训、融资、法律咨询等公共服务平台。加快建设国家玩具质量监督检验中心。办好玩具展、美食节、服装展、食博会等特色展会。积极发展旅游业，加快生态滨海旅游示范区建设，改善旅游环境，打造海滨休闲度假旅游品牌。

扩大消费。积极落实国家扩大内需、刺激消费的重大战略举措，大力推进商业网点规划建设，加快构建城乡双向互动的现代流通体系。推进卜蜂莲花等一批大型商贸服务项目建设。改善农村消费环境，提升农民消费水平。

　　（五）以帮助中小企业应对金融危机为出发点，促进中小企业健康发展

　　加大对中小企业的财税支持。落实国家、省扶持中小企业的各项税费优惠政策，研究出台促进中小企业健康发展的政策措施。在省产业引导资金中，拿出一定比例作为重点项目和龙头企业贷款贴息，以及配套省财政共建资金，扶持先进制造业、特色产业发展。设立中小企业发展专项资金，扶持企业技术改造、技术创新。

切实解决中小企业融资难问题。启动“金融支持工业园区建设”计划。搭建银企合作平台，引导金融机构改善服务，创新金融产品，简化贷款程序。设立中小企业信用担保基金，扶持信用担保机构发展。做好上市后备资源培育，加强分类指导，重点推进，继续扶持、推动一批企业上市，支持企业利用资本市场筹措资金。加快信用体系建设，营造良好金融环境。

促进中小企业转型升级。加强产学研和科技金融等合作创新，充分挖掘和运用国内外科研、人才和资本等资源，联合实施重大科技项目，开发核心技术和关键技术。以创建国家知识产权示范城市为契机，推动中小企业运用知识产权提升产业核心竞争力。鼓励企业创立自主品牌，提高产品市场占有率。推进公共科技创新平台建设，促进行业技术创新。加强特色产业基地、技术创新专业镇建设。

　　（六）以改革开放为动力，增强经济发展活力

　　加大力度扩大开放。争取广澳港区上半年通过国家一类口岸验收，抓紧申报完成汕头机场对外国籍飞机阶段性有限度开放。设立华侨投资产业园区，鼓励华侨和国内潮商回家乡投资兴业。申报综合保税区，推动保税区与广澳港区实施区港联动。以引进消化吸收再创新为着力点，集中力量突破重点领域核心技术，加速科技成果转化和产业化。推动对台海上货运直航可持续发展，争取开通与澎湖旅游、客运航线，建设南澳对台小额商品交易市场。

　　推动招商引资。把引进外资与调整产业结构、加快基础设施建设结合起来，重点引进石化、造船、电子信息、先进制造业和现代服务业项目，重点推介东部城市经济带、工业经济带、广澳和海门港区等基础设施项目。做好招商引资后续服务，提高签约项目履约率。加大对台招商力度。鼓励现有外资企业增资扩产，做强做大。

　　保持进出口稳定增长。制订出台激励外经贸发展政策措施。支持企业多生产国家政策鼓励的出口产品，动员企业抢抓出口订单。加强产品质量诚信体系建设，全面提高产品国际竞争力。引导加工贸易转型升级，加快发展服务贸易和技术贸易。培育和发展出口名牌，扩大高技术含量、高附加值产品出口。着力培育一批出口龙头企业，鼓励生产型企业自营出口。积极组织企业参加“广交会”及各类国际专业展会，巩固欧美日等传统市场，开拓新兴市场。扩大先进技术、关键设备和能源产品进口。建立和完善贸易摩擦应对机制，提高处理国际贸易争端的能力。开展共建文明口岸活动，完善口岸查验配套设施，推进大通关建设。

　　加强区域合作。主动参与海峡西岸经济区建设，积极争取国家优惠政策。办好闽粤赣十三市党政领导第十四次联席会议。巩固发展与港澳的合作关系。

　　加快国企改革重组。鼓励民营企业收购、兼并国有、集体企业。尽快完成市属国有企业、企业化管理事业单位的产权制度改革。

　　推进投融资体制改革。建立政府融资借用还良性循环机制，组建实施项目公司，尽快建设政府投融资平台。探索垄断行业和公用事业改革，选择一批市政设施和各类特许经营权进行招投标和公开拍卖。积极运用BT、BOT等模式，鼓励社会资本投资建设项目，形成多元化投融资体制。加强政府融资建设项目管理，提高合同贷款的到位率和贷款资金的使用效率。加快建筑业改革发展步伐，进一步打响外向型建筑业品牌。

　　（七）以精细化管理为着力点，不断优化发展环境

　　推进节能减排。落实节能减排工作责任制，建立完善考核体系，强化执法监察。鼓励发展能源资源消耗低、附加值高的产业，加快淘汰落后生产能力。开展资源综合和循环利用，重点支持能源资源节约和综合利用新技术、新工艺、新设备的研究开发。推行清洁生产，积极发展环保产业。推进重点领域和重污染行业的节能减排。

节约集约用地。继续推进土地利用总体规划修编。加快新溪、外砂片区土地规划修编。优化土地布局和结构，确保重点项目和产业建设用地需求。强化建设用地审批管理，严格控制新增建设用地，加强耕地特别是基本农田保护。加快清理盘活闲置和低效利用存量土地，提高土地节约集约利用水平。完善征地补偿制度，严格土地市场管理。加强土地执法，严厉查处土地违法违规行为，确保违法违规用地立案率、查处率、结案率等刚性指标的完成。

加强经济运行监测和财税管理。加强经济运行分析，深入研究关系发展全局的重大问题，及时跟踪并协调解决经济运行中出现的突出矛盾和问题。进一步完善市级财政管理体制，科学界定市、区县政府财权、事权，提高区县财政保障能力。积极培育税源，强化税收征管。规范非税收入管理。优化财政支出结构，把财力保障的重点进一步向社会事业、公共基础设施、生态建设、环境保护倾斜，向扩大就业、完善社会保障体系、改善困难群众生活倾斜。规范政府债务管理，妥善处理历史债务问题。结合事业单位分类改革，逐步推进政府购买服务。加快建立政府公共资源有偿使用机制，盘活运用城市公共资源。按年度财政一般预算收入1%的投入，全力援助草坡乡灾后恢复重建。

规范市场经济秩序。强化市场监管，依法打击制假售假、虚假广告等违法活动。大力开展产品质量和食品安全专项整治，全面整顿市场秩序，保障人民群众饮食和用药安全。强化反走私综合治理。推进企业信用体系建设，引导企业诚信经营。培育发展商会和行业协会。抓好市场供给，强化价格调控和监管手段，努力保持物价水平基本稳定。促进房地产市场健康发展。 加强城市管理和社会管理。启动河浦大道、泰山路、汕汾路绿化带建设，大力推进城乡绿化建设改造，全力实现创建国家园林城市工作目标。充分调动基层和市民的积极性，推进市容环境综合治理，加强“12319”热线建设，进一步完善城市管理、督察长效机制。推进交通综合执法改革，整治交通运输市场秩序，坚决打击非法营运行为。加强社会治安综合治理，提高路面见警率，加大执法力度，集中整治治安重点地区和突出问题，严厉打击“两抢一盗”、“黄赌毒”等违法犯罪活动，增强人民群众的安全感。积极发展社区服务，推进平安和谐社区建设。强化各级安全生产责任制，加大安全生产执法监察力度，突出抓好重点行业领域隐患排查治理，防范遏制重特大安全事故发生。高度重视信访工作，妥善处置群体性、突发性事件。

　　（八）以改善民生民安为重点，加快发展社会各项事业

坚持把为民办实事好事、改善民生作为政府工作的出发点和落脚点，进一步扩大公共财政覆盖范围，加大力度推进基本公共服务均等化，集中财力办好一批今年见效的民生实事，逐年解决关系人民群众切身利益的民生问题。

做好就业再就业工作。实施积极就业政策，建立健全城乡统一的就业管理体制和人力资源市场体系，尽快建立全市劳动力资源信息库。着力完善自主创业、自谋职业的扶持政策，发挥创业带动就业倍增效应。大规模开展职业技能培训，加快技能人才培养步伐，强化就业援助和服务。新增就业岗位6万个。

完善社会保障体系。扩大社会保险覆盖面，提高企业退休人员基本养老金，月人均增加73.1元。尽快解决我市早期离开国有、集体企业人员养老保障问题。以被征地农民养老保险制度为突破口，加快建立农村养老保险制度。完善城镇职工和居民基本医疗保险、新型农村合作医疗制度，不断提高医疗保障水平。健全社会救助体系，提高低保标准和补助水平，将农村家庭年人均纯收入低于1500元的家庭全面纳入低保。加强优抚安置和双拥工作，扶持发展妇女儿童、社会福利、老龄、慈善和残疾人事业。

大力发展教育人才事业。落实《汕头市基础教育投入保障条例》，加大教育投入。继续做好实施城乡免费义务教育工作，推动规范化学校建设，促进义务教育均衡发展。加快发展普高、中职和技工教育，在省补助资金基础上，市财政再投入1亿元加快学校基础设施建设，启动金中小礐石校区、鮀滨职业技术学校、纺织服装职业技术学校、市高级技工学校新校区建设，支持办好粤东高级技工学校。推进高等教育、成人教育稳步发展，继续办好汕头职业技术学院，支持办好汕头大学。进一步发展学前教育、特殊教育，规范扶持民办教育。改善老区、山区学校办学条件。加强教师队伍建设。认真落实有关政策，积极稳妥解决教师待遇和中小学代课教师问题。加强人才市场建设，优化用人环境，加大高层次、实用型人才引进力度，留住现有人才。

加强卫生医疗工作。深化医药卫生体制改革，解决群众看病就医问题。健全基层医疗卫生服务体系，改善县镇村三级卫生网络和城市社区卫生服务。设立贫困精神病人康复救助基金，建立精神病人防治救助长效机制。为200名贫困精神病人提供3个月免费住院治疗。改造完善市120急救指挥中心，改扩建市中心医院门诊急诊综合楼，启动市卫生学校易地重建和市中医医院原址扩建工程。加强卫生监督和疾病控制工作，及时处置各类突发公共卫生事件，保障人民群众身体健康。

继续推进文化体育等各项事业。深入开展公民道德教育，加强和改进未成年人思想道德建设，提高市民文明素质，推进省文明城市创建工作。加强公共文化服务体系建设，推进乡镇综合文化站和“农家书屋”建设，力争完成汕头戏曲学校重建。搞好第三次全国文物普查。大力发展动漫、文化创意等新兴文化产业。推进文化市场管理，重点抓好娱乐场所、网吧的监管和整治。广泛开展全民健身活动，加快体育设施建设，积极申办第十四届省运会。落实新修订的省人口计生条例，继续稳定低生育水平，提高出生人口素质。落实挂钩帮扶制度，推动濠江、潮阳、潮南三个后进地区加快转化。依法兑现计划生育奖励政策。巩固和提高火化率，落实殡葬管理目标责任制和目标考核制度。续建市新殡仪馆。推进五五普法教育工作。

解决群众切身利益问题。实施农村饮水安全工程，解决潮阳、潮南区26.76万人饮用水安全问题。实施中心城区“城中村”和涉农社区供水直抄到户改造工程，完成40个社区4.08万户居民直抄到户改造，实现用水同网同价。加快解决困难群众住房难问题，建设廉租住房669套，向3366户住房困难家庭提供租赁住房补贴，对1200户农村贫困危房户和300户农村泥砖房（茅草房）实施改造。发展城市公共交通，新开通公交线路4条，恢复路线3条，新增公交车辆60台，更新20台，翻新50台。建设农村客运线路10条、候车亭200个。加快农副产品市场建设，逐步解决中心城区东区买菜难问题。

　　（九）以提高行政效能为核心，加快建设服务型政府

严格依法行政。按照法定权限和程序履行职责，把依法行政贯穿于政府决策、执行、监督全过程，推进法治政府建设。完善重大问题集体决策、专家论证、社会听证和合法性审查等制度，推进行政决策科学化、民主化和制度化。重大决策主动听取人大及政协的意见建议，主动接受监督。认真落实人大及其常委会的各项决议决定，积极支持政协委员发挥参政议政作用，及时、高质办理人大议案建议和政协提案。加强政府法制工作，用好特区立法权和较大市立法权，不断推进各个领域的立法进程。强化行政执法监督，落实行政执法责任。加强行政复议工作。加强审计监督，确保中央和省扩大内需等资金用得快、用得准、用得实。进一步推进政务公开。强化公务员依法行政意识，提高依法行政能力和水平。

　　推进高效行政。实施地方政府机构改革工作，理顺政府部门职责关系。做好第四批行政审批制度改革。推进事业单位分类改革。加快电子政务建设，建立全市政府部门统一办公自动化系统，拓展网上受理、并联审批和一站式服务，以及网民参政议政的渠道和平台。提高行政服务中心事项入驻率和窗口直接办结率，进一步完善行政审批电子监察系统建设。完善应急管理机制，有效预防和处置各种突发事件。

保持廉洁行政。全面贯彻落实党风廉政建设各项规定，加强对领导干部、人财物管理使用、关键岗位的监督和制约。坚决纠正损害群众利益的不正之风，认真查处投资者和群众反映强烈的突出问题。加强经济责任审计，强化行政监督，坚决查处违法违纪案件，以清正廉洁取信于民。

切实转变作风。扎实推进精文简会，提高办文办事效率。坚持从实际出发，从人民群众利益出发，大兴调查研究之风，实事求是、求真务实，讲实话、重实干、办实事、求实效。坚持勤俭办一切事业，保持机关经费和业务经费零增长，防止讲排场、铺张浪费现象发生。

各位代表，全面转入科学发展轨道，争当实践科学发展观排头兵，是时代赋予我们的神圣使命。让我们高举中国特色社会主义伟大旗帜，紧密团结在以胡锦涛同志为总书记的党中央周围，在市委的正确领导下，团结依靠全市人民，开拓创新，扎实工作，为建设更加美好的汕头而努力奋斗！
